# A rare case of lingual mucosal leishmaniasis caused by reactivation of *Leishmania infantum* infection

**DOI:** 10.1186/s12879-025-10592-4

**Published:** 2025-02-18

**Authors:** Yannik Eggers, Martha Holtfreter, Irmela Müller-Stoever, Johannes Mischlinger, Andreas Hammacher, Bernhard Hemmerlein, Alexander Kreuter, Frank Oellig, Dennis Tappe, Tom Luedde, Torsten Feldt, Hans Martin Orth

**Affiliations:** 1 Department of Gastroenterology, Hepatology and Infectious Diseases, Medical Faculty University Hospital Düsseldorf, Heinrich-Heine-University Düsseldorf, Düsseldorf, Germany; 2 Center for Tropical Medicine, Bernhard Nocht Institute for Tropical Medicine & I. Department of Medicine University Medical Center Hamburg-Eppendorf, Hamburg, Germany; 3 German Center for Infection Research, Partner Site Hamburg-Lübeck-Borstel-Riems, Hamburg, Germany; 4 Klinik für Mund-, Kiefer- und Gesichtschirurgie, Helios Klinikum Duisburg-Homberg, Duisburg, Germany; 5 Institut für Pathologie, Helios Klinikum Krefeld, Krefeld, Germany; 6 Klinik für Dermatologie, Venerologie und Allergologie, Helios St. Elisabeth Klinik Oberhausen, Universität Witten/Herdecke, Oberhausen, Germany; 7 Institut für Pathologie, Mühlheim an der Ruhr, Germany; 8 Bernhard-Nocht-Institute for Tropical Medicine, Hamburg, Germany

**Keywords:** Mucosal leishmaniasis, *Leishmania Infantum*, Tongue, Cutaneous leishmaniasis, Reactivation

## Abstract

**Background:**

*Leishmania infantum* is the only prevalent *Leishmania* species in Europe and manifesting predominantly as cutaneous or visceral leishmaniasis, whereas new world species like *Leishmania* (*L.) braziliensis* are well known pathogens in mucocutaneous leishmaniasis. Mucosal leishmaniasis caused by *L. infantum* is a rare clinical condition with only few cases described in literature. In contrast to our case, mostly immunocompromised patients with no history of leishmaniasis are affected.

**Case presentation:**

We describe the case of a 77-year-old German male who developed an ulcerous lesion of the tongue. As oral cancer was suspected, the patient underwent surgery. After suspected diagnosis of *Leishmania* spp. in histopathology, the patient was referred to our department for further diagnostics and treatment. Relapse from a cutaneous leishmaniasis acquired in Spain is likely, as *L. infantum* could be identified as the causative agent. The patient recovered after treatment.

**Conclusions:**

Mucosal leishmaniasis caused by *L. infantum* is rare and usually mistaken for malignancy. As demonstrated, it can be preceded by cutaneous leishmaniasis of the immunocompetent. Due to possible dissemination systemic treatment should be applied.

## Introduction

In the Mediterranean region of Europe *Leishmania (L.) infantum* is the only prevalent species of the *Leishmania* family and manifesting predominantly as cutaneous (CL) or visceral leishmaniasis (VL) [1]. New world species from the Americas like *Leishmania braziliensis* are well known pathogens in mucocutaneous leishmaniasis (MCL) [2], while mucosal leishmaniasis (ML) caused by old-world species like *L. infantum* is a rare clinical condition with only few cases described in literature [3, 4]. However, a more recent study reports more frequent involvement of the mucous membranes in the oral and nasal cavities, which may hint at previous underdetection [5]. As far as detailed information is available, mainly elderly men are affected with a high proportion of immunocompromised patients [3, 4, 6–8]. Typically in ML, mucosal presentation is assumed to be the first manifestation of leishmaniasis and not accompanied by a history of previous CL or VL [4], however progression into VL has been described [4, 9]. It is debated, whether these locations represent the site of inoculation, or more likely are secondary to dissemination [8, 10]. Herein, we present the case of an immunocompetent male with ulcerating leishmaniasis of the lingual mucosa caused by *L. infantum* as a possible reactivation of a previous cutaneous leishmaniasis acquired in Spain.

## Case presentation

**Fig. 1:**
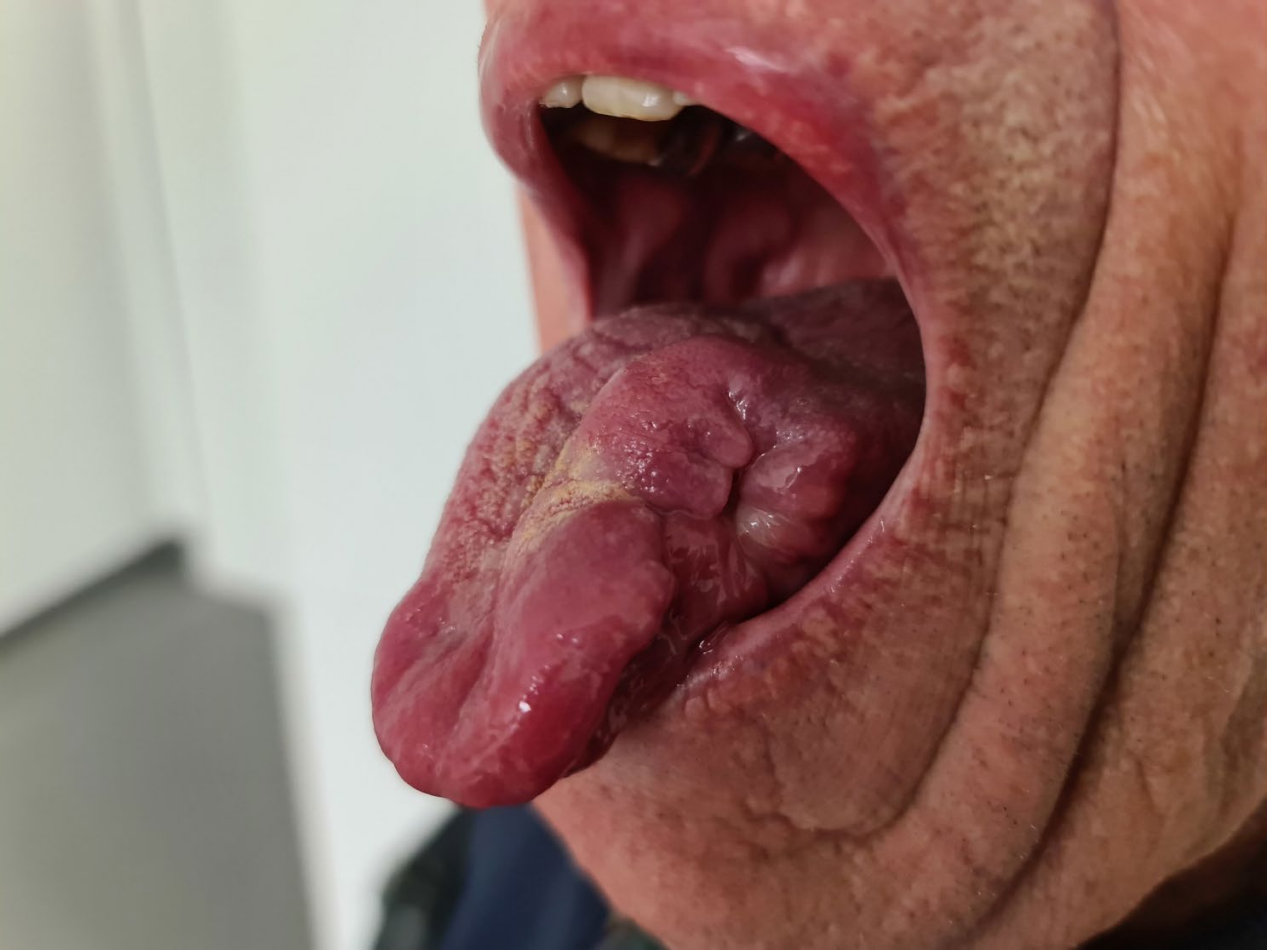
Clinical presentation of patient with a huge defect after surgery

**Fig. 2:**
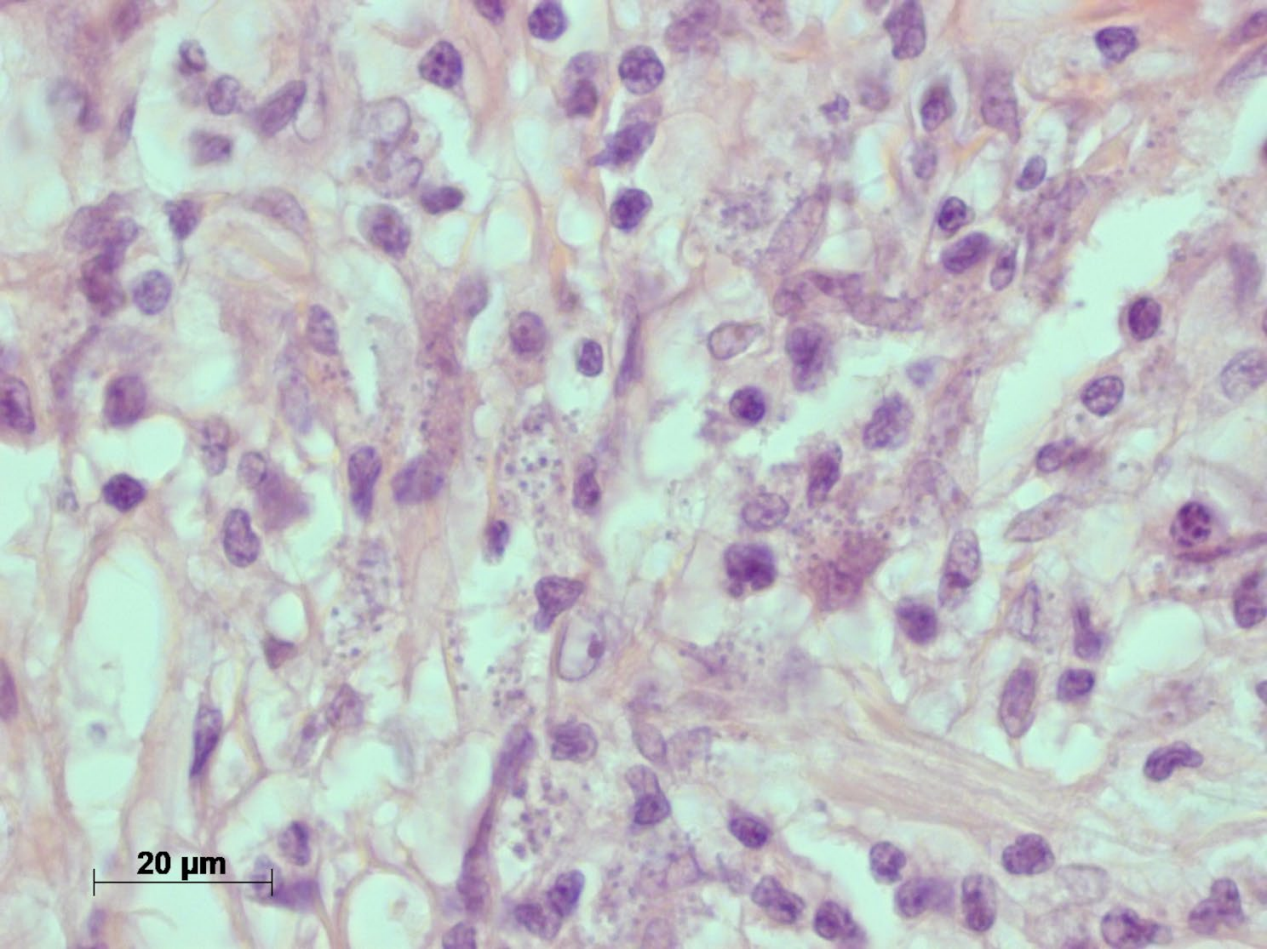
Histology of the lingual ulcer reveals amastigote forms of *Leishmania*

**Fig. 3:**
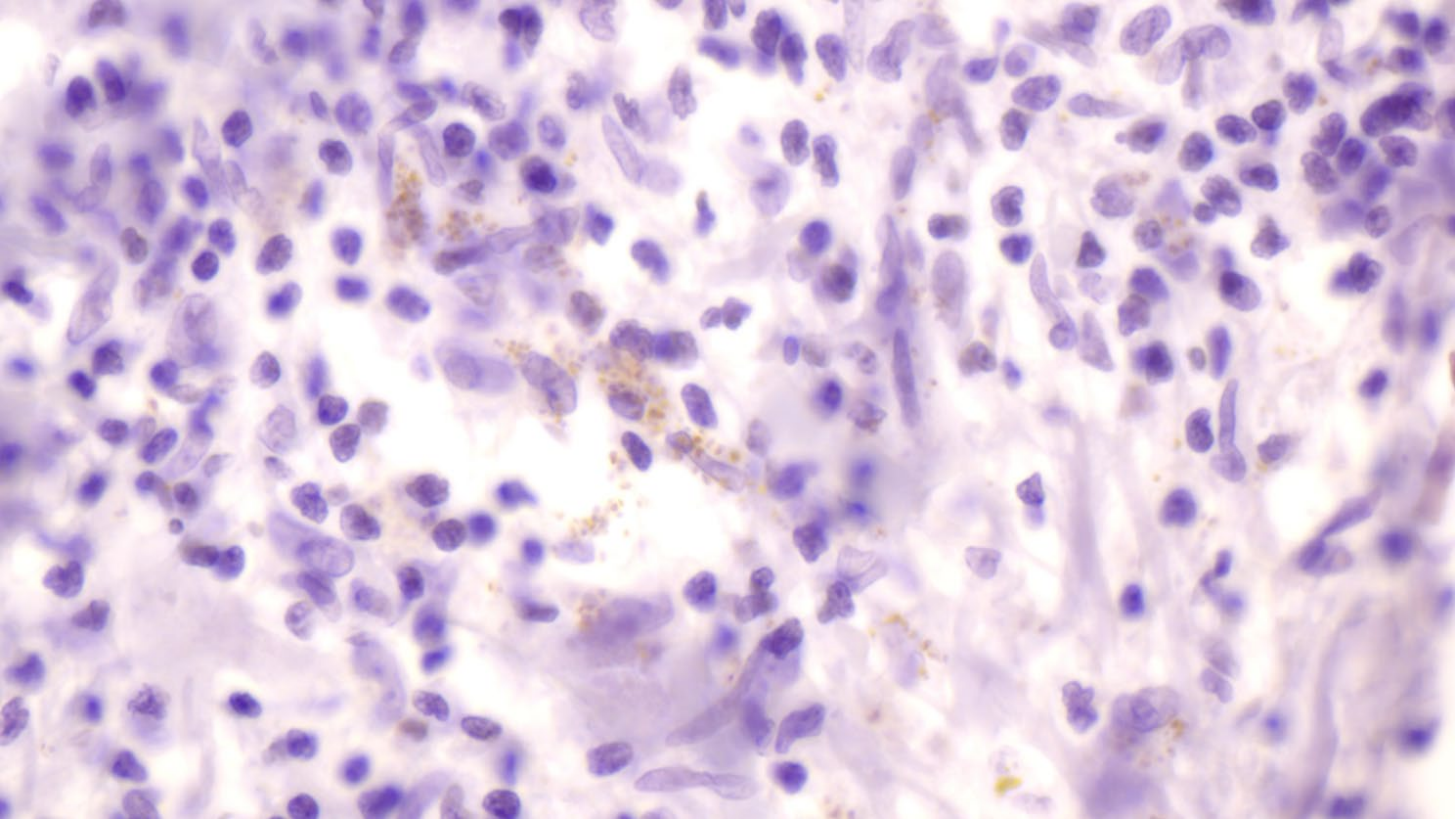
Immunostaining of cutaneous leishmaniasis with Anti-CD1a antibody (MTB1) highlighting amastigote forms of *L. infantum*

**Fig. 4:**
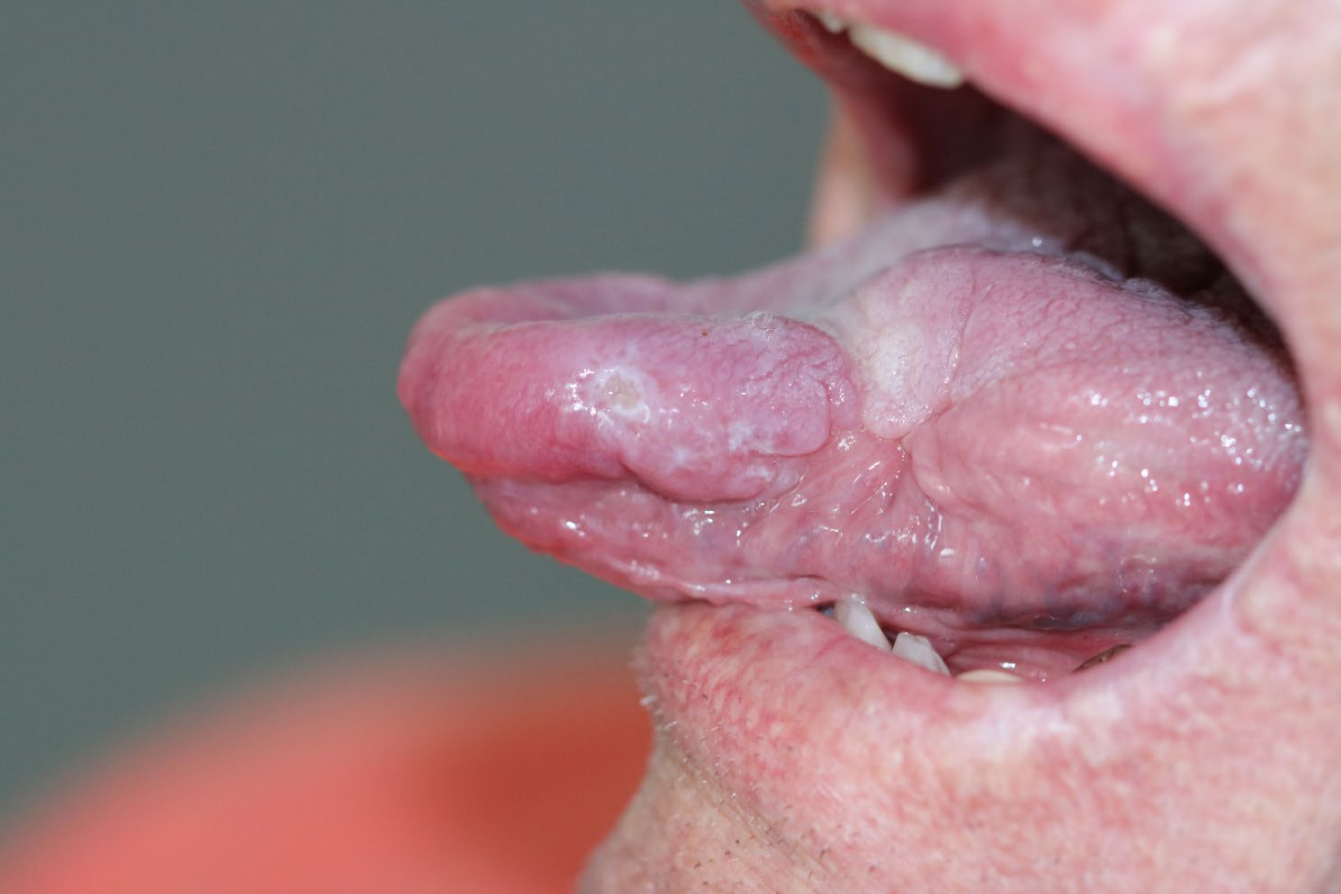
Small ulcerating lesion approximately 2 cm ventral to the resection site at clinical follow-up

A 77-year-old German male was referred to our tropical medicine department in April 2023 after oral surgery of a lingual ulcer clinically highly suspicious for malignancy (Fig. 1). Histopathologically, however, roundish intracellular structures could be detected which were compatible with amastigote Leishmania parasites (Fig. 2). During the past four months before presentation a single and slowly growing lesion was developing at the left distal third of his tongue causing halitosis and increasing difficulties in eating and speaking. There were no cervical, submandibular or nuchal lymph nodes palpable and no signs of hepatosplenomegaly. The patient did not report any fever, weight loss or night sweating. Erythrocytes, platelets and white blood count (WBC) were within the normal range. The WBC differential did not show an increase in eosinophils, but IgE levels were highly elevated (1440 IU/ml; normal < 100 IU/ml). Serum electrophoresis did not show an increase of the gamma fraction. Erythrocyte sedimentation rate (ESR) (28 mm/h; normal < 3–15 mm/h) and c-reactive protein (CRP) (2.7 mg/dl; normal < 0.5) were moderately elevated. Serological screening for *Treponema pallidum* (Chemiluminescent immunoassay, CLIA) and human immunodeficiency virus (HIV) was negative. There was no medical history besides arterial hypertension, obesity and an obstructive sleeping apnea syndrome (OSAS). The patient is a former smoker with the history of 40 pack years. He is retired and travelling occasionally to the Costa brava in Spain. No travel outside of Europe and no contact to animals was reported. For confirmation of the diagnosis and species identification scratching was performed carefully from the excision´s margin. Microscopy of the tissue smear did not show any amastigotes of *Leishmania* spp. in Giemsa staining. Nucleic acid amplification testing (NAAT) with a routine *Leishmania* complex-screening polymerase chain reaction (PCR) targeting the 18 S ribosomal ribonucleic acid (rRNA) genes [11] did not detect any *Leishmania* parasites. Serum antibodies against *Leishmania* species were detected by an in-house indirect immunofluorescence testing (IFAT) using cultured *L. infantum* and *L. major* promastigotes and fluorescence-coupled sheep anti-human IgGAM (total Ig). IFAT titer was 1:1,280 (normal < 1:20 as determined with 40 healthy blood donors), indicating a past or present infection. With a more sensitive PCR targeting the parasite’s kinetoplast deoxyribonucleic acid (DNA) [12], nucleic acid of *Leishmania* species was detected from the smear material. However, the amount of nucleic acid was too little to perform further PCRs used for sequencing and species identification from this material. Therefore, formalin-fixed paraffin-embedded (FFPE) tissue from the oral surgery was analyzed. Histology revealed amastigotes of *Leishmania* spp. (Fig. 2). From this material, the *Leishmania* complex-screening PCR [11] was positive for the *Leishmania donovani* complex and, following sequencing of the *cpb* gene [13], *Leishmania infantum* was identified (100% nucleotide homology with GenBank entry JN400125). This finding is in line with the travel history of the patient to northern Spain, although *L. infantum* is not known to frequently cause mucosal leishmaniasis. A second anamnesis revealed that the patient once was suffering from a badly healing wound on his right forearm eight years ago, but already forgot about it. At that time, the lesion was biopsied, excised and covered with a flap plastic surgery at a dermatology clinic. We therefore requested the dermatologist’s report and the archived FFPE material, which revealed the diagnosis of a cutaneous leishmaniasis in histopathology (Fig. 3). Following surgery, no species identification was performed and no sequential antiparasitic treatment was administered. The patient stayed asymptomatic until recently. On our microscopic examination from the stored slides, the diagnosis of CL was confirmed and PCR [11] detected *L. donovani* complex from the archived slides. Unfortunately, the nucleic acid quality was too poor and the amount too little to perform sequencing. *L. infantum* belongs to the *L. donovani* complex and is the only species of *Leishmania* species endemic in Spain [2], hence it can be assumed that the patient suffered from a reactivation of his cutaneous leishmaniasis eight years ago rather than re-infection by lingual inoculation. Due to the long travelling distance of the patient to our department and cost effectiveness, treatment was initiated with oral fluconazole 200 mg for six weeks. In the meantime, the patient was hospitalized due to an ischemic stroke. At clinical follow-up in October 2023 a new ulcerating lesion of the tongue approximately 2 cm ventral of the resection site of the previous mucosal leishmaniasis was noticed (Fig. 4). Again, *L. infantum* could be identified by PCR and sequencing [11, 13]. We started treatment with oral miltefosine 150 mg/d for 28 days. As of October 2024, no relapse was noticed.

## Discussion and conclusions

Leishmaniasis is an infectious protozoal disease caused by different *Leishmania* species and transmitted by a variety of sandflies from the *Phlebotomus* or *Lutzomyia* family [2]. There are an estimated 0.6 to 1 million cases of CL annually and 50,000 to 90,000 cases of VL respectively according to the WHO [1]. While VL and CL are well known conditions in old-world leishmaniasis, mucosal involvement has been described less frequently for *L. infantum* [3, 4, 10, 14]. Recently, a European multi-center study found higher rates of ML due to *L. infantum* than previously reported [5]. MCL by New World *Leishmania* species and ML by *Leishmania infantum* are different in their clinical appearance. MCL typically has a more nasal than oral tropism and usually starts at the nostrils or lips. Features of MCL are characteristic destructive lesions of the nasal septum, lips and palate, which can lead to permanent deformity like so-called tapir-nose and result in life-threatening conditions [2, 10]. ML from *L. infantum* however rather occurs in oral (lips, cheeks, palate, gums, tongue), pharyngeal or upper airway locations including the larynx and vocal cords [10, 14]. ML with oral and lingual involvement has been described more frequent in immunocompromised, but also occasionally in immunocompetent patients [4, 15, 16]. Different kinds of immunosuppressive conditions are known in ML [4], such as renal transplant [6, 8], cancer [7, 17, 18] and systemic or local immunosuppressive treatment including steroids and biologicals [10, 19]. Case series showed mainly elderly men are affected by ML [4]. A previous history of possible or confirmed CL or VL is rare [10]. Due to the scarcity of the disease, patients often are referred to oral surgery departments and evaluated for cancer [7, 10, 20]. Diagnosis can be delayed and sometimes patients receive inappropriate treatments [3]. Our case report describes localized mucosal leishmaniasis of the tongue in a patient with no underlying immunocompromising disease or treatment, which is a rare condition. There has been a debate, whether localized mucosal leishmaniasis by *L. infantum* represents the site of inoculation or is secondary to dissemination [8, 10]. As the amastigotes of *Leishmania* species can persist in macrophages for years after cutaneous inoculation, they can be reactivated in immunosuppressive condition and cause VL or ML subsequently [8]. Reactivation of leishmaniasis by *L. infantum* in immunocompetent patients is rare but has been described recently [9, 16]. This case report supports the hypothesis of secondary dissemination due to reactivation of leishmaniasis. It could be demonstrated that the patient was suffering from a previous CL by *L. donovani complex* acquired in Spain, where *L. infantum* is the only species of *Leishmania* present. Hence the preceding CL likely was reactivated and caused the current manifestation as ML, where *L. infantum* could by identified by sequencing. However, the patient continued to travel to Spain annually after the first diagnosis of cutaneous leishmaniasis in 2015 and therefore periodically was exposed to the risk of re-infection in a low-endemicity setting while Germany is non-endemic for *Leishmania* species. Although the patient suffered from obstructive sleeping apnea syndrome, inoculation of the parasites into the oral mucosa by *Phlebotomus* flies is unlikely, as OSAS is common in the European population but cases of oral leishmaniasis by *L. infantum* are rare and additionally, the second lingual lesion developed in Germany with no exposure to an endemic setting. However, also inapparent re-infection on other locations cannot be ruled out in general due to the patient´s travel history. After confirming the diagnosis of a previous CL from dermatohistopathology, we assumed dissemination of the parasites. The patient did not show any clinical or laboratory signs of VL caused by *L.* infantum. In particular, there was no fever, hypergammaglobulinemia or other elevated laboratory inflammatory markers. This is particularly relevant as VL typically exhibits hypergammaglobulinemia, while isolated ML and CL generally do not. Therefore, we decided against bone marrow aspiration (BMA). In literature, detection of *L. infantum* from BMA in ML varied [4, 7–9, 19] and PCR from EDTA (ethylenediaminetetraacetic acid) blood [21] often led to positive findings in healthy blood donors [22]. In our case the kinetoplast-PCR proved more sensitive as molecular screening method, as previously described [23]. However, the less sensitive complex-PCR has the advantage of narrowing down the pathogen to a complex in a single step [11]. The strategy of management can be discussed critically in this case. Miltefosine and liposomal amphotericin B are the most favored treatment options of old-world ML in literature [24, 25], but no evidence-based treatment option is available from randomized clinical trials. Miltefosine 150 mg/d for 28 days or liposomal amphotericin B (e.g. 3 mg/kg d1-5, 14, 21) have successfully been used for treatment of mucosal leishmaniasis due to *L. Infantum* [7, 10, 14, 20]. In literature, also successful systemic or intralesional treatment of mucosal lesions with pentavalent antimonials is mentioned [15]. However, given the classification of the lesion as complex and considering the history of a previous CL, we opted strongly against intralesional injections. Systemic antimony formulations are not favorable because of toxicity especially in elderly patients. Relapse is possible in ML and described for all standard treatment regimens. However, higher cure rates are reported for oral miltefosine than liposomal amphotericin B [24, 25]. In our case, despite low evidence, we considered oral fluconazole as a reasonable option in an out-patient setting, because miltefosine has high treatment costs and liposomal amphotericin B usually requires an in-patient setting, which both could not be handled by the patient and his family. Treatment failure with fluconazole may also be attributed to underdosing and discontinuation of the therapy after stroke. When suspecting dissemination of *Leishmania* from a previous lesion, although there were no signs of VL, oral miltefosine or intravenous liposomal amphotericin B rather than fluconazole should have been chosen as the drug of choice in the first round of treatment in this patient.

Our patient likely suffered from reactivation and dissemination of the parasites originating from a cutaneous leishmaniasis caused by *L. infantum* acquired in Spain eight years ago. However, re-infection cannot be fully ruled out due to the patient´s travel history. Our finding is significant, as published cases or case series of ML by *L. infantum* are usually not linked to a history of previous CL or VL. Additionally, the relapse of leishmaniasis in a non-immunocompromised patient eight years after first manifestation is remarkable. Diagnosis of ML is usually mistaken for malignancy. Therefore, in travelers from endemic regions ML caused by *L. infantum* should be considered in unclear mucosal lesions. Moreover, miltefosine should be considered as first line systemic treatment for old-world ML.

## Data Availability

All clinically relevant data have been made available within the manuscript. For data protection reasons, no further clinical data can be made available.

## Abbreviations

BMA: Bone marrow aspiration
CL: Cutaneous leishmaniasis
CLIA: Chemiluminescent immunoassay
CRP: C-reactive protein
DNA: Deoxyribonucleic acid
EDTA: Ethylenediaminetetraacetic acid
ESR: Erythrocyte sedimentation rate
FFPE: Formalin-fixed paraffin-embedded
HIV: Human immunodeficiency virus
L.: Leishmania
MCL: Mucocutaneous leishmaniasis
ML: Mucosal leishmaniasis
NAAT: Nucleic acid amplification testing
OSAS: Obstructive Sleep Apnea Syndrome
PCR: Polymerase chain reaction
rRNA: Ribosomal ribonucleic acid
VL: Visceral leishmaniasis
WBC: White blood count
